# BK Virus: Beyond Nephropathy Metastatic BK Virus-Induced, Donor-Derived Bellini’s Carcinoma in a Kidney Allograft Recipient: Boosting Rejection to Treat the Cancer

**DOI:** 10.3389/ti.2025.14664

**Published:** 2025-07-31

**Authors:** Flora Lefevre, Mélanie Benoit-Janin, Emilien Seizilles-de-Mazancourt, Xavier Matillon, Fanny Buron, Alice Koenig, Valérie Dubois, Matthieu Dietz, Olivier Rouvière, Emmanuel Morelon, Olivier Thaunat, Xavier Charmetant

**Affiliations:** ^1^ Centre of Nephrology and Renal Transplantation, Centre Hospitalo-Universitaire (CHU) Conception, Marseille, France; ^2^ Department of Pathology, Groupement Hospitalier Est, Hospices Civils de Lyon, Bron, France; ^3^ Department of Urology and Transplantation Surgery, Edouard Herriot Hospital, Hospices Civils de Lyon, Lyon, France; ^4^ Department of Transplantation, Nephrology and Clinical Immunology, Edouard Herriot Hospital, Hospices Civils de Lyon, Lyon, France; ^5^ French National Blood Service (EFS), Human Leukocyte Antigen (HLA) Laboratory, Decines, France; ^6^ Department of Nuclear Medicine, Groupement Hospitalier Est, Hospices Civils de Lyon, Bron, France; ^7^ Department of Urinary and Vascular Radiology, Edouard Herriot Hospital, Hospices Civils de Lyon, Lyon, France

**Keywords:** donor-derived carcinoma, BK virus BKPyV, BK virus derived carcinoma, alloimmune response, collecting duct carcinoma

Dear Editors,

BK virus (BKV), present in 80%–90% of the population, establishes a lifelong persistent infection in the kidney and urinary tract after a subclinical primary infection. It can reactivate and cause *de novo* infection in immunocompromised kidney transplant recipients (KTRs) lacking neutralizing antibodies against the donor strain [1], causing nephropathy (BKVAN) in 4%–8% of cases. Persistent BKV infection increases the risk of urothelial carcinoma and collecting duct carcinoma (CDC) [2].

A 73-year-old KTR was admitted for asthenia, acute kidney injury (creatinine 320 μmol/L), inflammatory syndrome (CRP 130 mg/L), and anaemia (Hb 75 g/L). He was followed for a KT performed 9 years earlier, complicated by biopsy-proven BKVAN at month 10. Mycophenolate mofetil was switched to everolimus (3–8 ng/mL), then to leflunomide, and tacrolimus to ciclosporin (80–120 ng/mL). The viral load decreased over 5 months and BKV was never detected again in the blood. At admission, MRI revealed a hypovascular mass in the graft with central necrosis and retroperitoneal inflammation. Biopsy confirmed a tumour composed of irregular tubular structures, trabeculae and single cells (Figure 1). The nuclei had a high mitotic index. Necrotic changes were observed. This tumour proliferation infiltrated between non-tumour and dysplastic premalignant tubules (Figure 1A). Immunohistochemistry showed diffuse positivity of tumour cells for PAX8, CK7, INI1, fumarate hydratase, and SDHB, and focal positivity for GATA3 (Figure 1B), but negativity for CK20, p504S, p63, or ALK. Only tumour cells showed strong nuclear staining with anti-SV40 large T-antigen (Figure 1C), leading to the diagnosis of BKV-associated CDC. No metastases were initially found, and transplantectomy was performed. On pathological examination, the tumour invaded the surgical margins of the transplantectomy. Immunosuppressive therapy was tapered by withdrawing leflunomide and reducing tacrolimus trough levels, but not entirely discontinued in order to minimize the risk of donor-specific alloimmunization. Two months later, PET/CT showed iliac, retroperitoneal, pelvic lymph node metastases, and a right ischiopubic bone metastasis. Bulk HLA genotyping of the biopsy revealed that the tumour was not of recipient origin. Immunosuppression was completely withdrawn to stimulate the allo-immune anti-tumoral response, and the patient achieved complete metastatic regression within 3 months. At 2 years, he remained recurrence-free.

**FIGURE 1:**
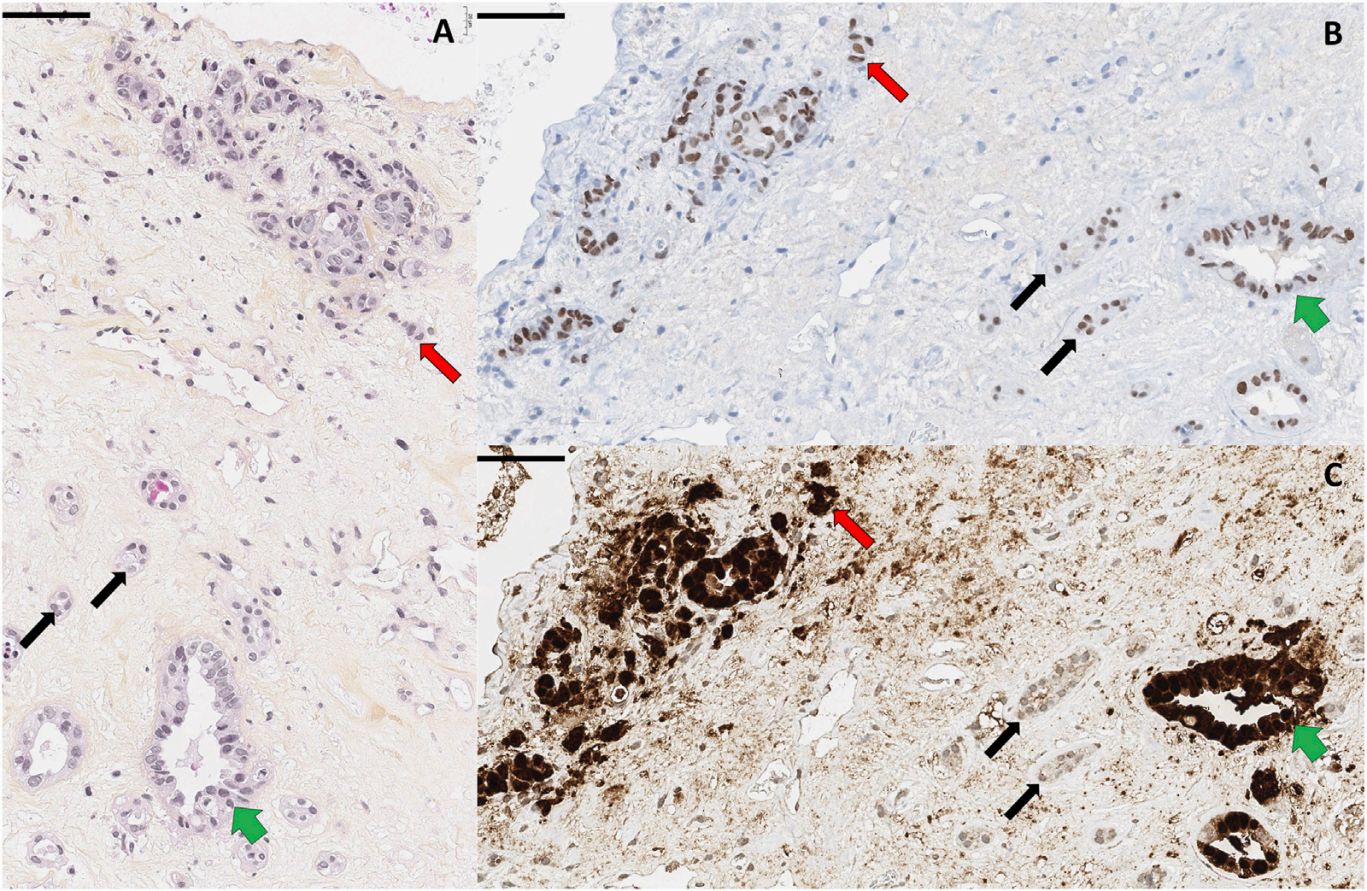
Pathological findings. **(A)** Haematoxylin-Eosin-Safran staining showing infiltrative carcinomatous cells (red arrows), dysplastic premalignant (green arrows) and normal (black arrows) renal tubules. **(B,C)** Immunohistochemical examinations showing PAX8 [renal origin, **(B)**] and Sv40 [viral antigen, **(C)**] labelling of the tumour. Scale bars 60 µm.

This is a very rare case of metastatic donor-derived BKV-induced CDC in a KTR, successfully managed without chemotherapy nor immunotherapy. Bellini’s CDC is a rare (<1%) and aggressive variant of renal cell carcinoma [2]. It has been hypothesized that CDC could be linked to BKV in transplanted patients [3]. No other specific risk factor have been identified. The tumorigenesis induced by BKV is known. Polyomaviruses encode 2 viral oncogenes, the small and the large T-antigen [4, 5]. They can inactivate tumour suppressor genes p53 and pRb. Deletion of p53 and pRB leads to gene instability and replication errors that contribute to oncogenesis. Dysregulation of large T-antigen, with persistent over-expression in non-lytic cells, promotes cell growth, genetic instability and neoplasic transformation [6, 7]. The high levels of large T-antigen expression in tumour nuclei is visualized by SV40 staining in immunohistochemistry. Microdissected samples of neoplasic cells usually contain DNA sequences specific for segments of BK-polyomavirus large T-antigen and VP1 genes. On the contrary, no BKV DNA sequences are detected in microdissected normal renal parenchyma [8]. Donor-derived tumours in KTRs are rare (<0.1%) and may arise from donor cells predisposed to oncogenesis. Key oncogenic drivers occur as early as late childhood and early adolescence. Then, late events during transplantation and under immunosuppression, such as BKV infection and genomic integration, may promote further oncogenesis in donor renal cells [9]. These donor-derived tumours offer a unique treatment opportunity: withdrawal of immunosuppression led to spontaneous alloimmune tumour rejection by enabling the immune system to target the graft through alloimmune and antitumour responses. Ortega *et al* reported remission of a metastatic donor-derived urothelial tumour after transplantectomy and immunosuppression withdrawal [10]. Meier *et al* achieved similar success in a metastatic Bellini carcinoma by boosting the anti-tumour immune response with IL-2 immunotherapy [3] (Supplementary Table S1).

This case highlights the specificity of urological tumours in KTRs. Identifying donor-derived malignancies may refine treatment strategies, reducing reliance on aggressive therapies. The clinical history reported in this case suggests pragmatic management, although this is by no means a recommendation. Firstly, given the very unfavourable prognosis of these tumours, it seems legitimate to perform surgery and completely stop immunosuppression. The two expected benefits of surgery are the removal of the largest possible tumour mass, and the avoidance of symptomatic toxic graft rejection. The addition of immunotherapy or chemotherapy should be discussed on a case-by-case basis, after evaluating the efficacy of the initial treatment. Given BKV’s oncogenic potential, long-term monitoring should extend beyond the risk of nephropathy to include surveillance for malignancy. Options could include annual urinary cytology screening, early invasive urological evaluation in the event of haematuria and potentially biannual imaging of the graft.

## Data Availability

The original contributions presented in the study are included in the article/Supplementary Material, further inquiries can be directed to the corresponding author.

## Supplementary Material

SUPPLEMENTARY TABLE S1 table summarizing post-transplant CDC cases linked to BK virus.
