# Supplementary material for: BK Virus: Beyond Nephropathy Metastatic BK Virus-Induced, Donor-Derived Bellini’s Carcinoma in a Kidney Allograft Recipient: Boosting Rejection to Treat the Cancer
Source: Transpl Int. 2025 Jul 31;38:14664. doi: 10.3389/ti.2025.14664 (PMC12351398; doi:10.3389/ti.2025.14664)
Supplement: Supplementary file 1 [file DataSheet1.pdf]

	Age (y)	Transplant	Time between transplantation and BKV (months)	Time between transplantation and CDC (y)	Time between BKV and CDC (y)	BK viremia at CDC diagnostic	Transplantectomy	Metastases	Immuno-suppression	Other treatment	Survival at last follow up
**This case**	73	Kidney	10	9	8	negative	yes	**yes** *2 months after transplan-tectomy*	stopped	**no**	yes at 2 years
**Meier et al**	41	Kidney + pancreas	24	9	7	negative	yes	**yes** *At the time of diagnosis*	stopped	IL2	yes at 6 years
**Dao et al**	39	Kidney + pancreas	24	11	9	negative	yes	no	continued	no	yes at 9 months
**Kenan et al**	62	Kidney	12	6	5	negative	yes	no	stopped	no	n.a.
**Veldhuijze et al**	62	Kidney	6	4,5	4	negative	yes	no	stopped	no	n.a.
